# Fibrates consistently lower risk of cardiovascular events across high-risk groups

**Published:** 2010-06

**Authors:** J Aalbers

**Affiliations:** Special Assignments Editor

## Introduction

A recent meta-analysis of published clinical trials conducted over the past 50 years has confirmed that the administration of fibrates to a broad range of high-risk patients lowers the risk of cardiovascular events.^1^

The 10 to 13% relative risk reduction (RRR) found in this meta-analysis could be regarded as modest, but in high-risk patients, this contribution to the prevention of cardiovascular events is significant. Fibrates showed no benefit in this meta-analysis on stroke, all-cause mortality or sudden death.

The cardiovascular event reduction was highest in patients with a higher mean baseline triglyceride concentration (> 2 mmol/l), a finding that agrees with subgroup analyses done in several clinical trials, including the ACCORD trial.^2^ No increased effect was seen in the subgroup of patients defined by a lower HDL cholesterol level in this metaanalysis. The ACCORD trial had placed patients with raised triglyceride and low HDL cholesterol levels in a subset, which showed greatest proportional risk reduction; such that only 20 individuals needed to be treated for five years to prevent one cardiovascular event.

Data from the ACCORD trial available in published form to the authors of the meta-analysis did not allow the low-HDL subgroup to be separated from the hightriglyceride subgroup. So currently, the role of fibrates in reducing cardiovascular events in a subgroup of patients with low HDL levels and triglycerides below 2 mmol/l should be the subject of further investigation.

This extensive meta-analysis included 45 000 individuals with a broad range of baseline characteristics. With regard to other cardiovascular conditions, the risk of heart failure was reported in three trials of 8 581 participants who had 584 heart failure events. Overall, there was no benefit of fibrates on heart failure. However, when the VA CO-OP Atherosclerosis trial,^3^ which included patients with pre-existing cerebrovascular disease, was excluded from the meta-analysis, fibrates showed an 18% reduction in heart failure.

Three trials including more than 15 000 patients showed that fibrates reduced the risk of albuminuria by 14%. A very significant reduction of 37% was seen in the relative risk of diabetic retinopathy in two trials, which included more than 10 000 patients.^4^,^5^
